# Prevalence of Urinary Incontinence in Female Professional Soccer Players by Category and Specific Position: A Comparative Study with a Control Group

**DOI:** 10.3390/healthcare12232478

**Published:** 2024-12-07

**Authors:** Julia M. Sebastian-Rico, María Jesús Muñoz-Fernández, Luis Manuel Martínez-Aranda, África Calvo-Lluch, Manuel Ortega-Becerra

**Affiliations:** 1 Physical and Sports Performance Research Centre, Faculty of Sports Sciences, Universidad Pablo de Olavide, 41013 Seville, Spain; jmsebric@alu.upo.es (J.M.S.-R.); acalllu@upo.es (Á.C.-L.); maortbec@upo.es (M.O.-B.); 2 Department of Physiotherapy, Francisco Maldonado University School, 41640 Seville, Spain; mariajmf@euosuna.org; 3 CTS 1110, UMSS Research Group, University of Seville, 41640 Andalusia, Spain; 4 SEJ-680: Science-Based Training (SBT) Research Group, Universidad Pablo de Olavide, 41013 Seville, Spain

**Keywords:** athletes, female, ICIQ-SF, pelvic floor dysfunctions

## Abstract

Background/Objectives: Urinary incontinence (UI) significantly impacts quality of life, with varying prevalence in women depending on factors such as age, childbirth, and type of sport practiced. This study compared the prevalence, types, and severity of urinary incontinence (UI) between professional female soccer players and sedentary students, analyzing its relation to playing position and competitive level. Methods: A descriptive, observational, and analytical cross-sectional study was conducted, assessing the prevalence, severity, and types of UI among 235 nulliparous professional female soccer players (experimental group, EG) and 252 sedentary female students (control group, CG). Data were collected using the short version of the International Consultation on Incontinence Questionnaire (ICIQ-SF). Statistical analyses included Fisher’s exact test to compare prevalence rates. Results: The findings revealed that 35% of soccer players and 31% of sedentary students reported experiencing UI. Stress urinary incontinence (SUI) was the most prevalent type in both groups, affecting 26% of soccer players and 14% of sedentary students, while mixed UI was more frequent among sedentary women (17%) (*p* < 0.05). No significant differences were observed in UI prevalence based on playing position or competitive level (*p* ≥ 0.05). However, female soccer players exhibited a significantly higher prevalence of UI during physical exertion or exercise compared to the control group (*p* ≤ 0.001), suggesting that high-impact sports may contribute to pelvic floor dysfunction. Additionally, 23.8% of soccer players reported mild-to-moderate UI severity. Conclusion: Female soccer players showed higher UI prevalence during exercise, underscoring the need for targeted interventions like pelvic floor training.

## 1. Introduction

Urinary incontinence (UI) is defined as any involuntary leakage and objectively demonstrable urine loss, constituting a significant social and hygienic problem for the person experiencing it, leading to a substantial impact on their quality of life [1]. Currently, three common types of urinary incontinence are recognized in the female population. Stress urinary incontinence (SUI) is associated with involuntary urine leakage triggered by physical exertion (e.g., coughing or sneezing). Urge urinary incontinence (UUI) is characterized by involuntary urine loss that follows a strong, often uncontrollable urge to urinate. Mixed urinary incontinence (MUI) combines involuntary urine loss related to both urgency and exertion [2].

The performance of women’s sports has experienced significant growth in recent decades. The physical and physiological characteristics of female athletes require specific preparation associated with the discipline and level of competition. However, sports practice is considered a risk factor for the presence of urinary incontinence (UI) in young female athletes [3]. This condition affects between 20% and 50% of women [1], with prevalence rates also varying from 25% to 45% depending on the population studied and the diagnostic criteria used [4]. While UI is influenced by various factors such as age, parity, and physical activity, it is more common in women who have undergone pregnancies, experienced childbirth with pelvic floor muscle tears, and sustained ligamentous and fascial injuries [5,6,7].

The prevalence also varies depending on the type of sport practiced. High-impact sports are one of the risk factors for UI in women [8]. Sport disciplines such as gymnastics, trampolining, and athletics are associated with increased prevalence rates of UI due to the repetitive increase in intra-abdominal pressure that challenges pelvic floor function. This can lead to a delayed response in blocking the contraction of pelvic floor muscles on command due to alterations in muscle fibers as a result of years of sports practice and their consequent impact [3,9,10,11]. Some authors mention that each individual may have a threshold, referring to the intensity and volume of training, beyond which the pelvic floor musculature cannot withstand repetitive impact [12]. This highlights the need to further investigate sport-specific factors contributing to UI, particularly in female soccer players, a group exposed to intense physical activity with potentially high pelvic floor demands.

Several studies have examined this condition in female athletes, both professional and amateur. In the realm of endurance sports, it was found that 50% experienced minor urine loss, with 27.6% associated with UUI and 45.4% with SUI [13]. Rodríguez-López et al. [14] reported the prevalence of UI in elite female Spanish athletes to be 51.7%, highlighting the necessity of early detection of UI and the implementation of training routines to improve and prevent this condition. Additionally, they emphasized that SUI showed the highest prevalence in their study sample. The prevalence of UI has also been reported in female CrossFit athletes, being higher in those over 35 years old with previous pregnancies and vaginal deliveries [15]. In soccer, studies are scarce, with none having been found with professional soccer players or concerning categories and playing positions. Only one study has reported on the prevalence of UI in amateur adolescent players aged 12 to 19 years [16].

Other studies on UI show heterogeneous results regarding the prevalence of this disorder in female athletes, mainly due to the disparity in selection criteria for the study samples [7,17]. However, there seems to be consensus on the use of the International Consultation on Incontinence Questionnaire—Short Form (ICIQ-SF), which is the most commonly used tool for assessing UI in adults [8,9,18,19,20], endorsed by the International Continence Society (ICS) [21,22].

The scarcity and heterogeneity of previous studies indicate the need to expand knowledge about UI and its types, and how categories and playing positions could affect the appearance of this disorder in female professional soccer players due to the different demands that the game places on the athletes. Therefore, this research had the following objectives: (i) to determine and compare the prevalence of urinary incontinence in female soccer players competing professionally in the Spanish women’s soccer leagues (First and Second Leagues), taking into account playing position and comparing UI prevalence with that of young, sedentary female students; and (ii) to assess the type of incontinence most frequently occurring in the aforementioned groups.

## 2. Materials and Methods

### 2.1. Design

A descriptive, observational, and analytical cross-sectional study was designed involving female professional Spanish soccer players from the First and Second divisions and young, sedentary female students. This study was conducted following the fundamental principles of the Declaration of Helsinki, the European Council Convention on Human Rights and Biomedicine, the Universal Declaration of UNESCO on the Human Genome and Human Rights, the Convention for the Protection of Human Rights and Dignity of the Human Being concerning the Applications of Biology and Medicine [22], and Spanish Data Protection Law [23]. The Ethics Committee of Pablo de Olavide University (Seville, Spain) approved this study with code 24/5-1.

### 2.2. Participants

The participant group in this research consisted of female soccer players (*n* = 235) from the Spanish First and Second Leagues (active players from the 2022–2023 season), representing diverse geographic regions across Spain, with a mean age of 25.7 ± 4.7 years, and young, sedentary students (*n* = 252) with a mean age of 20.6 ± 4.3 years. Sedentary students were recruited from various universities to provide a representative control group. They were classified as sedentary based on self-reported physical activity levels. Specifically, participants were asked if they engaged in regular physical activity or sports (defined as more than one hour per week of structured exercise or sports training). Only those who indicated they did not meet this criterion were included in the sedentary group. This classification ensured that the control group represented individuals with low physical activity levels.

Soccer players were categorized into four standard playing positions—goalkeepers, midfielders, wingers, forwards, and defenders—based on their primary roles during matches. Participants self-reported their positions, ensuring accurate representation of their playing roles.

A representative population of all players who met the inclusion criteria was considered, within a 95% confidence interval and a 5% margin of error (*p* ≤ 0.05). All participants were invited to take part voluntarily and anonymously in this research by email. They were asked to complete the ICIQ-SF questionnaire during March 2024. Before completing the ICIQ-SF, informed consent documents had to be read and accepted. Participants under 18 years of age required permission from their legal guardians to participate in this study. Double responses were eliminated by analyzing participants’ registered email addresses.

The inclusion criteria were as follows: female professional soccer players and students over 16 years of age, with no history of pregnancy or childbirth (nulliparous) who were able to understand the questionnaire in Spanish and had no neurological condition that made it difficult to comprehend the questionnaire. Exclusion criteria were participants who had undergone pelvic floor surgery. Participants were also not allowed to be involved in other sporting activities during the study. All participants were informed of the study’s objectives, questionnaire instructions, and the possibility of public access to the information once the research was completed.

### 2.3. Procedures

Data were collected through self-administered questionnaires during March 2024, based on the ICIQ-SF [4,24], developed by the ICS, and translated and validated for the Spanish language [25,26]. The scoring of the ICIQ-SF determines the presence or absence of UI by summing the scores of questions 3, 4, and 5, classified as follows: mild, 1 to 5 points; moderate, 6 to 12 points; severe, 13 to 18 points; and very severe, 19 to 21 points [26,27,28]. The fourth question in the questionnaire serves as a descriptive and indicative measure of the UI type. The presence of SUI was determined by answering the question “When do you lose urine?” with a positive response of “during physical exertion/exercise” and/or “when coughing or sneezing”. UUI was identified if participants answered positively to when they lose urine to “leaks before you can get to the toilet”. MUI was identified as a combined positive response of previously identified SUI and UUI. All responses were considered if they resulted in a score greater than 0 on the ICIQ-SF.

Specific characteristics (age, playing position, and level of competition) were recorded via questions added to the original ICIQ-SF. The ICIQ-SF questionnaires were completed by participants independently to ensure privacy and reduce potential bias. A member of the research team was present to provide assistance or clarification if needed, ensuring that all questions were understood correctly. Once completed, the questionnaires were collected and reviewed for completeness by the research team. Data were manually entered into a secure database by trained personnel. Double-checking procedures were employed to minimize errors during data entry. Numerical scores from the ICIQ-SF were calculated according to the official guidelines, ensuring consistency and accuracy in assessing the severity and impact of urinary incontinence.

Participants’ BMI was also calculated based on self-reported height and weight data, and playing position (goalkeepers, midfielders, wingers, forwards and defenders) and competitive level (First or Second Spanish League) were recorded for soccer players as previously mentioned. While these data were included to describe the population, BMI was not directly analyzed in relation to UI prevalence, and no additional details regarding years of playing experience or training intensity were collected.

### 2.4. Statistical Analyses

Descriptive statistics were used based on the mean (m) and standard deviation (SD). When appropriate, data are presented as percentages. Statistical significance was established at the *p* ≤ 0.05 level. The distribution of each variable was examined using the Kolmogorov–Smirnov normality test. The homogeneity of variance was verified using Levene’s test. When necessary, and when the data did not follow a normal distribution, non-parametric tests were used, which justified the use of the Mann–Whitney U test to compare differences between groups. Additionally, the chi-square test was employed to analyze associations between categorical variables, such as the prevalence of UI across groups or categories. The Kruskal–Wallis test was used for within-group mean contrasts. Analyses were performed using SPSS software, version 21.0 (SPSS Inc., Chicago, IL, USA), and the free statistical package JASP (Version 0.9.2; University of Amsterdam, The Netherlands).

## 3. Results

The characteristics of the observational group (OG) are presented in Table 1. The data are organized by taking into account the level of competition and playing positions.

Table 2 presents the prevalence of urinary incontinence (UI) within the study sample, along with age and ICIQ-SF scores across different groups and subcategories. The observational group (OG) showed a slightly higher UI prevalence (35%) compared to the control group (CG), which reported a prevalence of 31%. Despite this difference, no statistically significant variations were observed in ICIQ-SF scores between the two groups (*p* = 0.405). When examining subcategories within the OG, such as competitive level and playing position, no significant differences were found in UI prevalence. Specifically, the prevalence ranged from 39% in the First Spanish League to 29.9% in the Second Spanish League (*p* = 0.283). Similarly, comparisons by playing position did not yield statistically significant differences (*p* = 0.895).

Based on the scores obtained in the ICIQ-SF questionnaire, the severity of UI was determined in the study sample as shown in Table 3. None of the participants in the OG or CG experienced very severe UI (a score above 18).

The data obtained from question 4 of the ICIQ-SF show the perceptions of each subject with regard to the reasons they associate with urine loss. Regarding SUI, we found a prevalence of 26% for the OG and 14% for the CG. No significant differences were found in the reasons between the OG and the CG, except for “leaks when you are physically active/exercising”, which did show a significance level of *p* < 0.001; and for “leaks when you have finished urinating and are dressed” (*p* ≤ 0.05) (Table 4).

The presence of UUI was 10% for the OG and 14% for the CG with no significant differences between groups. Only 4.7% of the OG and 1.6% of the CG showed MUI according to their self-reported answers in the ICIQ-SF.

## 4. Discussion

This study investigated the prevalence of UI among female professional soccer players in Spain, comparing the results by playing position and with a CG of sedentary young women, as well as identifying the most common types of incontinence in these groups. The results obtained in this research show that professional female soccer players in Spain have a 35% prevalence of UI, as measured through the ICIQ-SF questionnaire. Additionally, athletes exhibited a higher percentage of mild severity (23.8%), with SUI being the most common type (26%) in our study sample. The prevalence of UI in the CG was 31%, with SUI (14%) being the most prevalent.

Comparing the prevalence of UI in female soccer players (35%) with young students (31%), we observed that the CG had a lower incidence of this condition. Even though there were no significant differences between these prevalences, our data follow previous studies where 24.6% of nulliparous women attending a gym presented with UI compared to 14.3% in a CG [8]. In contrast to these studies, some authors reported less incontinence in physically active women compared to sedentary women but highlighted the difficulty of interpreting it because women with UI may have stopped exercising [16,29,30,31,32].

Although both study groups showed higher values of SUI, there were significant differences in the reasons associated with it (*p* < 0.001). Players reported urine loss during physical activity (15%) at a higher rate than the student group (4%). Bo et al. [5] found similar SUI data comparing elite athletes (41%) with women (39%) of similar average age. Although no significant differences in SUI were found between the two groups, athletes had higher levels of urine loss during physical activity. These authors highlight the high prevalence of healthy, young, nulliparous women with UI (39.4%).

One important fact to highlight in our research is that no significant differences were found in the prevalence of UI, or the severity or possible type of the same, depending on the position in which participants were playing on the field. Rodriguez-Lopez et al. [14] presented differences between the prevalence of UI, taking into account the event of specialization in athleticism. This suggests that the physical and physiological demands of specific positions would not influence this condition for female soccer players. However, it would be appropriate to analyze whether the type of training and exercise to be performed, as well as its intensity, taking into account its impact, could affect the presence of UI in female soccer players.

According to the type of sport performed, a systematic review included 7507 women aged 12 to 69 years [7]. The prevalence of UI was 42.22% in basketball, 50% in soccer, and 61% for gymnastics, all considered high-impact sports. Regarding moderate impact, the prevalence was 31.58% in hockey, 44.44% in judo, and 31.02% in running. Lower prevalences were found in low-impact sports such as cycling (10.11%), Pilates (5.56%), and swimming (15.25%). The data obtained in our research may differ from those provided in this study due to the women included in soccer (*n* = 38) and the large heterogenicity of the studies selected. Soccer is considered a high-impact sport because it involves jumps, sprints, and rotational movements, with impact on the ground. This suggests that the increase in abdominal pressure could modify the morphology and functionality of ligaments and connective tissue, which is related to the main cause of UI in young nulliparous women with no other risk factors [33].

The prevalence of urinary incontinence (UI) observed in this study among professional female soccer players (35%) is considerably lower than the 62.8% reported in the study by Filoni et al. [16], which examined adolescent amateur soccer players. Several factors may explain this discrepancy. Firstly, differences in competitive level and associated training regimens could play a crucial role. Adolescent players may lack the structured training and professional guidance available to professional athletes, potentially leading to less effective pelvic floor muscle engagement and conditioning. Furthermore, the amateur status of the athletes in Filoni et al.’s study introduces variability in training routines and physical preparedness, factors that might exacerbate UI prevalence.

Secondly, the developmental and physical maturity of participants may contribute to these differences. Adolescent players, aged 12–19 in the study of Filoni et al. [16], may be at a stage where the pelvic floor musculature is not fully matured, increasing their susceptibility to stress-related UI. In contrast, the professional players in the present study were older (mean age of 25.7 years), likely benefiting from more advanced musculoskeletal development and experience in managing the physical demands of their sport.

Lastly, the methodological differences between the studies must be considered. Filoni et al. [16] used both the ICIQ-SF and the pad test to assess UI, while the present study relied solely on the ICIQ-SF. This broader diagnostic approach in the 2018 study may have captured a higher prevalence of UI symptoms. Moreover, the smaller sample size and amateur setting of Filoni et al.’s study may have increased the influence of individual variability.

Despite these differences, both studies underscore the significant prevalence of UI in female soccer players. This highlights the need for targeted interventions, such as pelvic floor muscle training (PFMT), to prevent and manage UI in athletic populations. Future research should adopt longitudinal designs to explore the interplay of age, training intensity, and pelvic health, providing a more comprehensive understanding of UI in this demographic.

Furthermore, Hagosvska et al. [19] analyzed SUI, identifying an average value of 11.9% of SUI in 278 women from 10 different sports: fitness (15.6%), athletics (23.8%), basketball (14.7%), volleyball (19.6%), handball (16.6%), soccer (5%), and tennis, figure skating, and dance (0%). Considering the UI values obtained for soccer, these authors concluded that the sample size (*n* = 20) could make these values inconclusive for the referred sport. SUI values in basketball, volleyball, and handball athletes were similar to those obtained in the current research (15%). In the same vein, trampoline gymnasts have the highest UI values (>70%) [11,21,34]. These authors agree that although they are expected to have adequate muscle tone to prevent UI, it seems that their pelvic floor muscles do not respond continently during trampoline landings.

Additionally, a UI prevalence of 51.7% (*n* = 109) was found in Spanish athletics, with SUI (64.4%) being the most common among the athletes analyzed. Athletes specializing in vertical jumps had a lower prevalence than those performing horizontal jumps in their training and competition [14]. These differences around athletic disciplines seemed not to be present in the soccer sample in our study because there were no differences between playing positions. This result suggests that physical demands [35] may not interfere with UI in young, nulliparous female soccer players.

So far, the most common type of UI in young nulliparous women is SUI. However, data obtained in our investigation showed 10% in the OG and 14% in the CG of UUI, along with MUI in 4.7% of the OG and in 1.6% of the GC, which need to be considered in evaluating the presence of UI in this population of young women. Do Santos et al. [36] observed a 48% prevalence of UI, although with contrary data regarding types: 50% UUI, 37.5% SUI, and 12.5% MUI. This study reported that hours of training were a risk factor for UI. The differences around the types of UI data described could be explained by the huge range of sports modalities, in contrast with the homogeneity of our study sample.

The data obtained in our research follow the trend of previous studies, where SUI has the highest prevalence in female athletes [6,11,13,14,20,27]. UI is present in athletes in both endurance sports such as cross-country skiing and running [13], and in strength sports [15].

In the present investigation, our data show that there is a considerable presence of UI in female soccer players, with no differences in comparison with the general population with no previous delivery event. This suggests the importance of taking into account pelvic health in women [37] in terms of prevention training.

Many athletes do not report urine loss during training or competition [38]. Some authors mention possible causes for the presence of UI in this population, such as weak connective tissue combined with high-intensity, high-impact exercises [34,39]. Additionally, UI poses a hygienic and social problem [40] for those athletes who suffer from it [10], as well as imposing limitations on sports activity [41].

This study has several limitations that should be considered when interpreting the results. Firstly, the cross-sectional design limits our ability to establish causality between urinary incontinence (UI) and factors such as training intensity, playing position, or age. Longitudinal studies would provide greater insight into the progression and potential causes of UI among female soccer players. Secondly, the reliance on self-reported data from the International Consultation on Incontinence Questionnaire—Short Form (ICIQ-SF) introduces a potential for recall bias or underreporting. While the ICIQ-SF is a validated tool widely used in research, future studies could benefit from incorporating objective diagnostic measures, such as the pad test or urodynamic evaluations, to complement self-reported data and provide a more comprehensive assessment of UI.

Additionally, this study focused exclusively on nulliparous female athletes, which limits the generalizability of the findings to women with different obstetric histories or to male athletes. Comparative studies examining populations with diverse backgrounds and physiological profiles would enhance understanding of UI prevalence and its risk factors across different athletic groups.

The relatively small sample size, especially when stratified by playing position, may have reduced the statistical power to detect differences within subgroups. Future research should consider larger, more diverse samples to allow for robust subgroup analyses and the exploration of variables such as training intensity, match frequency, or injury history. Additionally, this study’s sample composition presents some limitations. While efforts were made to ensure geographic and socioeconomic diversity, detailed demographic data were not collected. Moreover, the discrepancy between the average age of the soccer players (25.7 years) and the sedentary students (20.6 years) reflects the recruitment focus, as professional soccer players are typically active in their mid-to-late twenties, while the sedentary group comprised undergraduate students. Although age differences may influence pelvic floor health, the nulliparity of all participants minimized confounding factors related to obstetric history. This age difference is acknowledged as a limitation since age is a potential factor in pelvic floor health. Future research should aim to minimize such discrepancies and include additional demographic data to provide a more comprehensive understanding of urinary incontinence in both athletic and non-athletic populations.

Finally, in addition, this study did not include detailed data on soccer players’ years of playing experience or training intensity, which may influence the prevalence of UI. Additionally, while BMI data were collected, they were not analyzed in relation to UI prevalence, which represents an area for further investigation in future studies. Also, the absence of data on participants’ PFMT history or awareness of pelvic health could be a notable limitation. Incorporating these variables into future research could help identify potential protective factors and guide the development of targeted prevention and intervention programs.

Future studies should prioritize longitudinal designs, include both subjective and objective measures of UI, and explore the effects of specialized training programs on pelvic floor health. Additionally, examining the impact of hormonal factors, such as the menstrual cycle, and broader psychosocial variables, such as stigma or quality of life, would provide a more holistic understanding of UI in athletic populations. Furthermore, while the ICIQ-SF addresses the general impact of UI on quality of life, it does not capture sports-specific challenges or psychological effects and coping strategies during practice and games. Future studies should consider incorporating sports-focused assessments or qualitative interviews to better understand the influence of UI on athletic performance and participation.

## 5. Conclusions

This study aimed to investigate the prevalence of urinary incontinence (UI) among professional female soccer players and sedentary young women, as well as to identify the most common types of UI and examine potential associations with playing position and competitive level. The findings reveal that more than one-third of professional female soccer players reported experiencing UI, with stress urinary incontinence (SUI) being the most prevalent type. In the control group, one-third of participants reported UI, with a lower prevalence of SUI.

Importantly, no differences in UI prevalence or severity were observed based on playing position or competitive level among the athletes. However, female soccer players exhibited a higher prevalence of UI during physical exertion or exercise compared to sedentary women, emphasizing the potential impact of high-impact sports on pelvic floor health.

These findings highlight the importance of addressing pelvic health in female athletes and integrating PFMT into regular training programs to mitigate the effects of UI. Future research should focus on longitudinal studies, larger sample sizes, and the inclusion of objective diagnostic measures to better understand the long-term implications of UI in sports performance and quality of life.

## 6. Practical Implications and Recommendations

### 6.1. Pelvic Floor Training Programs

Given the high prevalence of urinary incontinence (UI) observed in this study, particularly stress urinary incontinence (SUI) among soccer players, we recommend the implementation of tailored PFMT programs. These programs should be specifically designed for high-impact athletes to address the unique physical demands of their sports. Regular PFMT sessions, supervised by a trained physiotherapist, could improve pelvic floor muscle strength, endurance, and coordination, potentially reducing the incidence and severity of UI. Athletes could benefit from integrating these exercises into their pre-season conditioning routines and maintaining them throughout the competitive season.

### 6.2. Early Screening for UI in Sports Settings

Proactive screening for UI during routine sports medical evaluations could help identify at-risk athletes early. Incorporating validated tools like the ICIQ-SF into annual check-ups or pre-season assessments could facilitate the early detection of UI symptoms, even in athletes who may not report them spontaneously due to stigma or lack of awareness. Early identification allows for timely intervention, preventing the progression of symptoms and minimizing their impact on athletic performance and quality of life.

### 6.3. Educational Programs and Interdisciplinary Collaboration

Educating athletes, coaches, and medical staff about the prevalence of UI in high-impact sports and the benefits of PFMT can help reduce stigma and promote open discussions about pelvic health. Awareness campaigns and workshops could be implemented in sports organizations to normalize conversations around UI and encourage athletes to seek support.

Collaboration between sports physicians, physiotherapists, and trainers is essential to developing and implementing effective UI prevention strategies. An interdisciplinary approach ensures that interventions are holistic and tailored to the specific needs of athletes, combining physical, psychological, and educational support.

## Figures and Tables

**Table 1:** Descriptive variables of female soccer players.

**Competitive Level**	**First Spanish League**		**Second Spanish League**		**Observational Group**	
	***n***	**%**	***n***	**%**	***n***	**%**
	128	54.5	107	45.5	235	100
**Playing Position**	**First Spanish League**		**Second Spanish League**		**Observational Group**	
	***n* = ** **128**	**%**	***n* = ** **107**	**%**	***n* = ** **235**	**%**
Defender	42	32.8	29	27.3	71	30.3
Forward	24	18.8	19	18	43	18.4
Winger	14	10.9	18	17	32	13.7
Midfielder	37	28.9	26	24.2	63	26.8
Goalkeeper	11	8.6	15	14.2	26	11.1

*n* = total of participants.

**Table 2:** UI prevalence, age, and ICIQ-SF scores (mean ± SD) obtained.

		*n*	Age	UI Prevalence	%	ICIQ-SF Score
Group	Control	252	20.6 ± 4.3	78	30.9	4.6 ± 2.5
	Observational	235	25.7 ± 4.7	82	34.9	4.6 ± 2.4
Competitive level (OG)	First Spanish League	128	28.0 ± 5.0	50	39	4.4 ± 2.3
	Second Spanish League	107	26.5 ± 4.1	32	29.9	4.9 ± 2.7
Playing position (OG)	Defender	71	27.5 ± 4.4	25	35.2	4.8 ± 3.1
	Forward	43	28.1 ± 5.3	14	32.5	4.3 ± 2.1
	Winger	33	26.5 ± 3.6	11	33.3	4.1 ± 2.5
	Midfielder	62	27.2 ± 4.9	27	43.5	4.8 ± 2.5
	Goalkeeper	26	26.8 ± 5.2	5	19.2	5.0 ± 1.8

*n* = total of participants; SD = standard deviation; UI = urinary incontinence; ICIQ-SF: International Consultation on Incontinence Questionnaire—Short Form; OG: observational group.

**Table 3:** UI severity in female soccer players and in controls.

Variable	Control Group (*n* = 252)		Observational Group (*n* = 235)		Total (*n* = 487)	
UI Severity	*n*	%	*n*	%	*n*	%
Mild UI (1–5 points)	60	23.8	59	25.2	119	24.4
Moderate UI (6–12 points)	17	6.7	22	9.4	39	8
Severe UI (13–18 points)	1	0.4	1	0.4	2	0.4
Very severe UI (up to 18 points)	0	-	0	-	0	0

*n* = total of participants; UI = urinary incontinence.

**Table 4:** Self-perception of urine leakage.

Variable	CG (*n* = 252)		OG (*n* = 235)		
UI Self-Perception (ICIQ-SF 4th Question)	*n*	%	*n*	%	*p* Value
Never—urine does not leak	186	74	165	70	0.262
Leaks before you can get to the toilet	36	14	24	10	0.121
Leaks when you cough or sneeze	26	10	25	11	0.889
Leaks when you are asleep	3	1	0	-	-
Leaks when you are physically active/exercising	9	4	36	15	<0.001 **
Leaks when you have finished urinating and are dressed	13	5	3	1	0.012 *
Leaks for no obvious reason	11	4	13	6	0.683
Leaks all the time	0	-	2	1	-

*n* = total of participants; ICIQ-UI-SF = International Consultation on Incontinence Questionnaire—Short Form; CG = control group; OG = observational group; * *p* ≤ 0.05 statistical differences; ** *p* ≤ 0.001 statistical differences. Note: Data do not have to be cumulative, as multiple responses could be selected.

## Data Availability

Data are available upon request to the contact author.

## References

[1] Robles J. (2006). La Incontinencia Urinaria. Urinary Incontinence. An. Sist. Sanit. Navar..

[2] González-Ruiz de León C., Pérez-Haro M.L., Jalón-Monzón A., García-Rodríguez J. (2017). Actualización En Incontinencia Urinaria Femenina. Semer. Med. Fam..

[3] Joseph C., Srivastava K., Ochuba O., Ruo S.W., Alkayyali T., Sandhu J.K., Waqar A., Jain A., Poudel S. (2021). Stress Urinary Incontinence Among Young Nulliparous Female Athletes. Cureus.

[4] Espuña Pons M., Castro Díaz D., Carbonell C., Dilla T. (2007). Comparación Entre El Cuestionario “ICIQ-UI Short Form” y El “King’s Health Questionnaire” Como Instrumentos de Evaluación de La Incontinencia Urinaria En Mujeres. Actas. Urol. Esp..

[5] Bø K., Borgen J.S. (2001). Prevalence of Stress and Urge Urinary Incontinence in Elite Athletes and Controls. Med. Sci. Sports Exerc..

[6] Cardoso A.M.B., Lima C.R.O.P., Ferreira C.W.S. (2018). Prevalence of Urinary Incontinence in High-Impact Sports Athletes and Their Association with Knowledge, Attitude and Practice about This Dysfunction. Eur. J. Sport Sci..

[7] de Mattos Lourenco T.R., Matsuoka P.K., Baracat E.C., Haddad J.M. (2018). Urinary Incontinence in Female Athletes: A Systematic Review. Int. Urogynecol. J..

[8] Fozzatti C., Riccetto C., Herrmann V., Brancalion M.F., Raimondi M., Nascif C.H., Marques L.R., Palma P.P. (2012). Prevalence Study of Stress Urinary Incontinence in Women Who Perform High-Impact Exercises. Int. Urogynecol. J. Pelvic Floor Dysfunct..

[9] Da Roza T., Brandão S., Mascarenhas T., Jorge R.N., Duarte J.A. (2015). Urinary Incontinence and Levels of Regular Physical Exercise in Young Women. Int. J. Sports Med..

[10] Alves J., Da Luz S., Brandão S., Da Luz C., Jorge R., Da Roza T. (2017). Urinary Incontinence in Physically Active Young Women: Prevalence and Related Factors. Int. J. Sports Med..

[11] Eliasson K., Larsson T., Mattsson E. (2002). Prevalence of Stress Incontinence in Nullipanous Elite Trampolinists. Scand. J. Med. Sci. Sport..

[12] Eliasson K., Edner A., Mattsson E. (2008). Urinary Incontinence in Very Young and Mostly Nulliparous Women with a History of Regular Organised High-Impact Trampoline Training: Occurrence and Risk Factors. Int. Urogynecol. J..

[13] Poświata A., Socha T., Opara J. (2014). Prevalence of Stress Urinary Incontinence in Elite Female Endurance Athletes. J. Hum. Kinet..

[14] Rodríguez-López E.S., Acevedo-Gómez M.B., Romero-Franco N., Basas-García Á., Ramírez-Parenteau C., Calvo-Moreno S.O., Fernández-Domínguez J.C. (2022). Urinary Incontinence Among Elite Track and Field Athletes According to Their Event Specialization: A Cross-Sectional Study. Sport. Med. Open.

[15] Álvarez-García C., Doğanay M. (2022). The Prevalence of Urinary Incontinence in Female Crossfit Practitioners: A Systematic Review and Meta-Analysis. Arch. Esp. Urol..

[16] Filoni E., Fitz F.F., Silva A., Filho J.M. (2018). Evaluation of the prevalence of urinary incontinence symptoms in adolescent female soccer players and their impact on quality of life. J. Yoga Physio..

[17] Hampel C., Artibani W., Espuña-Pons M., Haab F., Jackson S., Romero J., Gavart S., Papanicolaou S. (2004). Understanding the Burden of Stress Urinary Incontinence in Europe: A Qualitative Review of the Literature. Eur. Urol..

[18] Pastore A.L., Palleschi G., Al Salhi Y., Riganelli L., Fuschi A., Autieri D., Petrozza V., Carbone A. (2016). Evaluation of Sexual Function and Quality of Life in Women Treated for Stress Urinary Incontinence: Tension-Free Transobturator Suburethral Tape versus Single-Incision Sling. J. Women’s Health.

[19] Hagovska M., Švihra J., Buková A., Dračková D., Švihrová V. (2018). Prevalence and Risk of Sport Types to Stress Urinary Incontinence in Sportswomen: A Cross-Sectional Study. Neurourol. Urodyn..

[20] Rebullido T.R., Gómez-Tomás C., Faigenbaum A.D., Chulvi-Medrano I. (2021). The Prevalence of Urinary Incontinence among Adolescent Female Athletes: A Systematic Review. J. Funct. Morphol. Kinesiol..

[21] Donovan J., Abrams P., Cardozo L., Khoury S., Wein A. (2006). Symptom and Quality of Life Assessment. Incontinence: Basics and Evaluation.

[22] Consejo de Europa (1997). Convenio Para La Protección de Los Derechos Humanos y La Dignidad Del Ser Humano Con Respecto a Las Aplicaciones de La Biología y La Medicina (Convenio Relativo a Los Derechos Humanos y a La Biomedicina). Bol. Of. Del Estado.

[23] Jefatura del Estado (2018). Ley Orgánica 3/2018, de 5 de Diciembre, d e Protección de Datos Personales y Garantía de Los Derechos Digitales. Boletín Of. Del Estado.

[24] Avery K., Donovan J., Peters T., Shaw C., Gotoh M., Abrams P. (2004). ICIQ: A Brief and Robust Measure for Evaluating the Symptoms and Impact of Urinary Incontinence. Neurourol. Urodyn..

[25] Vicente E., Barrio M., Gual J., Fadil Y., Capdevila M., Muñoz J., García D., Hannaoui N., Prats J. (2016). Spanish (Spain) Validation of a Specific Symptomatic Questionnaire for Male Patients With Nocturia. Neurourol. Urodyn..

[26] Espuña-Pons M., Rebollo-Álvarez P., Puig-Clota M. (2004). Validación de La Versión Española Del International Consultation on Incontinence Questionnaire-Short Form. Un Cuestionario Para Evaluar La Incontinencia Urinaria. Med. Clin..

[27] Molina-Torres G., Moreno-Muñoz M., Rebullido T.R., Castellote-Caballero Y., Bergamin M., Gobbo S., Hita-Contreras F., Cruz-Diaz D. (2023). The Effects of an 8-Week Hypopressive Exercise Training Program on Urinary Incontinence and Pelvic Floor Muscle Activation: A Randomized Controlled Trial. Neurourol. Urodyn..

[28] Klovning A., Avery K., Sandvik H., Hunskaar S. (2009). Comparison of two questionnaires for assessing the severity of urinary incontinence: The ICIQ-UI SF versus the incontinence severity index. Neurourol Urodyn..

[29] Danforth K., Townsend M., Lifford K., Curhan G., Resnick N.M., Grodsteinr F. (2006). Risk Factors for Urinary Incontinence among Middle-Aged Women. Am. J. Obstet. Gynecol..

[30] Zhu L., Lang J., Wang H., Han S., Huang J. (2008). The Prevalence of and Potential Risk Factors for Female Urinary Incontinence in Beijing, China. Menopause.

[31] Schettino M.T., Mainini G., Ercolano S., Vascone C., Scalzone G., D’Assisi D., Tormettino B., Gimigliano F., Esposito E., Di Donna M.C. (2014). Risk of Pelvic Floor Dysfunctions in Young Athletes. Clin. Exp. Obstet. Gynecol..

[32] Wohemani R., Sullivan J., Jayakaran P. (2021). A Systematic Review of Information Brochure on Stress Urinary Incontinence for Female Athletes in Papua New Guinea. Contemp. PNG Stud..

[33] Shaw J.M., Nygaard I.E. (2017). Role of Chronic Exercise on Pelvic Floor Support and Function. Curr. Opin. Urol..

[34] Almeida M., Barra A., Saltiel F., Silva-Filho A., Fonseca A., Figueiredo E. (2016). Urinary Incontinence and Other Pelvic Floor Dysfunctions in Female Athletes in Brazil: A Cross-Sectional Study. Scand. J. Med. Sci. Sport..

[35] Marcelli L., Silvestri F., Di Pinto G., Gallotta M.C., Curzi D. (2024). How Match-Related Variables Influence the Physical Demands of Professional Female Soccer Players during the Regular Season. J. Funct. Morphol. Kinesiol..

[36] Dos Santos K.M., Da Roza T.H., Da Silva L., Wolpe E., Da Silva G., Tonon da Luz S. (2018). Female Sexual Function and Urinary Incontinence in Nulliparous Athletes. Phys. Ther. Sport.

[37] Ojedo-Martín C., Rodríguez-López E.S., Acevedo-Gómez M.B., Úbeda-D’Ocasar E., de-Diego M.V., Lara B. (2024). At What Point in the Menstrual Cycle Are the Pelvic Floor Muscles at Their Weakest?. J. Funct. Morphol. Kinesiol..

[38] Goldstick O., Constantini N. (2014). Urinary Incontinence in Physically Active Women and Female Athletes. Br. J. Sports Med..

[39] Bø K., Sundgot-Borgen J. (2010). Are Former Female Elite Athletes More Likely to Experience Urinary Incontinence Later in Life than Non-Athletes?. Scand. J. Med. Sci. Sport..

[40] Bradway C., Dahlberg B., Barg F.K. (2010). How Women Conceptualize Urinary Incontinence: A Cultural Model. J. Women’s Health.

[41] Coyne K., Sexton C., Irwin D., Kopp Z., Kelleher C., Milsom I. (2008). The Impact of Overactive Bladder, Incontinence and Other Lower Urinary Tract Symptoms on Quality of Life, Work Productivity, Sexuality and Emotional Well-Being in Men and Women: Results from the EPIC Study. BJU Int..

